# Limitations of venous thromboembolism risk assessment models in medical patients: Response to “Unmet definitions in thromboprophylaxis for hospitalized medical patients: An appraisal for the need of recommendation”

**DOI:** 10.1016/j.rpth.2022.100027

**Published:** 2023-02-15

**Authors:** Chaozer Er, Alexander T. Cohen

**Affiliations:** 1 Department of General Medicine, Woodlands Health, Singapore, Singapore; 2 Department of Haematological Medicine, Guy’s and St Thomas’ NHS Foundation Trust, London, United Kingdom

*Dear Editor,*

We read with interest the recent review of Ferreira and colleagues describing imprecise and unclear areas in thromboprophylaxis for hospitalized medical patients [1]. However, we consider that there are other important uncertainties in this field, particularly in relation to limitations of available risk assessment models (RAMs).

An ideal RAM is properly derived in a suitable population and externally validated to accurately identify the risk benefit of initiating thromboprophylaxis after factoring in both the risks of venous thromboembolism (VTE) and bleeding [2,3]. Its use should lead to improvements in thromboprophylaxis rates and reduced hospital admission–related VTE [3]. It should be cost effective and simple to use [3]. Current RAMs have the following limitations:

## Derivation Issues

Many RAMs have been derived in populations of patients already receiving thromboprophylaxis [4]. This can lead to underestimation of the true risk of VTE in high-risk groups that are most likely to receive thromboprophylaxis and overestimation of relative risk in those not receiving thromboprophylaxis [4]. Many RAMs, such as the Padua prediction score (PPS), Geneva risk score, and Kucher model, were derived empirically based on experts’ opinion and literature reviews [3]. Many were studied in populations that included 40% to 61% of at-risk patients receiving thromboprophylaxis [[5], [6], [7], [8]]. Hence, the scores recognized a significant proportion of patients in whom thromboprophylaxis had failed rather than those at risk.

## Low Sensitivity

We agree with you that both PPS and the International Medical Prevention Registry on Venous Thromboembolism (IMPROVE) score have modest ability to predict the risk of VTE [1]. However, the Medically Ill Patient Assessment of Rivaroxaban versus Placebo in Reducing Post-Discharge Venous Thrombo-Embolism Risk (MARINER) study applied a modified IMPROVE risk score to identify medical patients who may benefit from extended thromboprophylaxis [9]. VTE occurred only in 1.1% of patients in the placebo group, which is much lower than the estimated 2.5%, consistent with IMPROVE RAM not identifying a high-VTE–risk population [9]. The Prevention of Venous Thromboembolism Disease in Emergency Departments (PREVENU) study found that Caprini score, IMPROVE score, or PPS did not perform better than simply using a patient’s advanced age alone for VTE risk assessment [10]. Furthermore, a study showed that none of the RAMs had both high sensitivity and specificity [11]. The authors advised that clinical judgment should supplement RAM for VTE risk assessment [11].

## Ease of Use

RAMs require the assessment of many risk factors. Caprini RAM includes 39 variables and, hence, is time consuming and difficult to use [3]. We agree with Ávila Ferreira et al. [1] that mobility assessment in patients can be challenging and subjective given the different definitions of reduced mobility and the timing of assessment of mobility. Initial postadmission assessment of mobility is generally used; however, mobility may deteriorate throughout the course of admission.

## High Risk of Bias

One systematic review, using Prediction model Risk Of Bias ASsessment Tool (PROBAST), a prediction model risk of bias assessment tool, examined 51 studies related to 24 VTE RAMs in adult hospitalized patients and showed that all studies had either a high risk of bias or insufficient information to estimate the bias [4]. The authors found that VTE RAMs had weak predictive accuracy [4]. The lack of methodologic clarity in most studies made it difficult to assess applicability [4]. The authors concluded that there is insufficient evidence to recommend 1 RAM over another, which is consistent with previous systematic reviews [[2], [3], [4]].

## Lack of Bleeding Risk Assessment

Other than the IMPROVE bleeding score, which was separately devised by the IMPROVE workgroup, all RAMs do not include bleeding risk assessment.

## Underrepresentation of Certain Groups

Many high-risk patients, such as those who are obese or have chronic kidney or liver disease, are either not recorded or are underrepresented in original studies [12]. The 2022 International Society on Thrombosis and Haemostasis guideline concurred that there is insufficient evidence to support the use of VTE RAMs to guide thromboprophylaxis in hospitalized patients with cirrhosis [13].

In conclusion, current RAMs have many limitations. We recommend developing a RAM from populations with minimal bias and from populations that are unaffected by treatments that alter actual risks. These populations are available from placebo-controlled randomized trials and observational data of untreated populations, including those from the prethromboprophylaxis era. Removing the risk of bias and the effect of thromboprophylaxis in the study population would allow a fresh start for accurate assessment of risk.
